# Compact Gut Microbial Dysbiosis Signature Associated with Necrotizing Enterocolitis and Altered Early Microbiota Development

**DOI:** 10.3390/pathogens15080785

**Published:** 2026-07-24

**Authors:** Ying Xiang, Zhuoqi Zhao, Zhong Feng, Jialu Zhuang, Yu Chen, Yansong Chen, Shumin Feng, Liya Pan, Zhilong Yan, Li Hong

**Affiliations:** 1 Department of Laboratory Medicine, Shanghai Children’s Medical Center, Shanghai Jiao Tong University School of Medicine, Shanghai 200127, China; 2 Department of Clinical Nutrition, Shanghai Children’s Medical Center, Shanghai Jiao Tong University School of Medicine, Shanghai 200127, China; zhaozhuoqi@scmc.com.cn; 3 Department of General Surgery, Shanghai Children’s Medical Center, Shanghai Jiao Tong University School of Medicine, Shanghai 200127, China; leosu24@163.com; 4 Department of Neonatology, Shanghai Children’s Medical Center, Shanghai Jiao Tong University School of Medicine, Shanghai 200127, China; zhuangjialu@scmc.com.cn; 5 Department of Laboratory Diagnostics, KingMed Center for Clinical Laboratory, Shanghai 200120, China; sh-chenyu@kingmed.com.cn (Y.C.); cys997522@163.com (Y.C.); sh-fengshumin@kingmed.com.cn (S.F.)

**Keywords:** necrotizing enterocolitis, gastrointestinal microbiome, dysbiosis, machine learning, infants, premature, biomarkers

## Abstract

Background: Necrotizing enterocolitis (NEC) is a life-threatening intestinal disorder in preterm infants and is strongly associated with gut microbial dysbiosis. However, whether recurrent genus-level dysbiosis patterns can be observed across heterogeneous NEC cohorts and summarized as a compact, interpretable microbial signature remains unclear. Methods: We analyzed two public neonatal gut microbiome cohorts and an independent real-world cohort within a three-stage framework of discovery, contextual analysis, and external validation. Community composition, alpha diversity, beta diversity, and differential genera were evaluated using harmonized genus-level features within each cohort. Machine learning feature prioritization was used to derive a reduced NEC-associated microbial signature. A four-group cohort was then used to examine this signature in relation to disease status, antibiotic exposure, and early postnatal development. The reduced signature was finally examined in our collected samples. Results: NEC-related microbial alterations showed marked heterogeneity at the whole-community level across cohorts, whereas several genus-level directional patterns recurred across datasets. Across datasets, NEC was repeatedly associated with enrichment of several opportunistic genera, including *Enterobacter*, *Klebsiella*, *Serratia*, and *Escherichia–Shigella*, and depletion of commensal or probiotic-associated taxa such as *Bifidobacterium*, *Lactobacillus*, and *Pediococcus*. Feature prioritization yielded a compact microbial signature that improved discrimination over the unfiltered abundance profile in the discovery cohort (AUC 0.674 vs. 0.59). In the four-group cohort, the NEC_ABT group remained distinct from healthy controls, supporting partial modification rather than normalization of the NEC-associated pattern. In our collected samples, the signature retained directional consistency but showed only modest external discrimination (AUC 0.618). Conclusions: These findings identify recurrent genus-level dysbiosis features associated with NEC across clinically heterogeneous settings. The compact microbial signature may serve as an exploratory biomarker for longitudinal and mechanistic validation, but not as a standalone diagnostic tool.

## 1. Introduction

Necrotizing enterocolitis (NEC) remains one of the most devastating gastrointestinal emergencies in neonatal intensive care and primarily affects very low birth weight (VLBW) and extremely preterm infants. A global systematic review and meta-analysis of VLBW infants showed that NEC continues to occur at clinically significant rates across regions, with substantial inter-center variability that complicates risk prediction and performance benchmarking [1]. Mortality remains high, particularly among infants who require surgical intervention, and survivors often face long-term sequelae, including short bowel syndrome and neurodevelopmental impairment, underscoring the importance of earlier recognition and prevention [2,3]. Despite decades of investigation, early NEC commonly presents with nonspecific manifestations that overlap with feeding intolerance and sepsis, whereas radiographic abnormalities may appear only after intestinal injury has progressed [4,5]. This diagnostic uncertainty can lead to delayed treatment escalation in some infants and overtreatment in others, including prolonged empiric antibiotic exposure and unnecessary interruption of enteral feeding [6,7]. There is therefore a need for biomarkers that reflect disease-relevant biology before catastrophic deterioration and remain robust across clinical heterogeneity and center-specific practice patterns [8,9]. Microbiome profiling has emerged as a promising approach because NEC is closely associated with microbial colonization patterns in the preterm gut, although the translation of microbiome-derived signals into reproducible clinical tools remains difficult [5,10].

Multiple independent studies suggest that NEC is more often preceded by intestinal dysbiosis than by a single causative pathogen, with a typical pattern of expansion in gram-negative taxa and depletion of putatively beneficial anaerobes [4,5]. A systematic review and meta-analysis of pre-NEC microbiome studies found an increased relative abundance of *Proteobacteria* before disease onset, supporting dysbiosis as a temporally antecedent feature rather than a secondary consequence of intestinal necrosis [10]. Early culture-independent studies also identified disease-associated stool microbial patterns, supporting the view that community-level alterations may carry clinical relevance even when no single pathogen is reproducibly detected [11]. Even so, published findings remain only partly consistent across cohorts, reflecting both the biological heterogeneity of NEC and substantial variation in sampling time, sequencing methods, and analytic pipelines [5,10]. Antibiotic exposure is a major source of confounding. Early empiric antibiotic use can markedly alter the infant gut microbiota and has been associated with NEC risk in several populations, although the direction and magnitude of this association vary across settings and exposure definitions [6,7,12]. Postnatal age and developmental stage also shape trajectories of gut colonization, so analyses that do not account for age-dependent microbial dynamics may obscure genuine disease-related signals [13]. These considerations indicate that microbiome-based NEC markers should be evaluated in study designs that explicitly disentangle disease effects from those related to antibiotic exposure and developmental stage [8,9]. An additional unresolved question is whether NEC is better reflected by recurrent directional dysbiosis patterns at the genus level than by a uniform shift in overall community structure.

From a translational standpoint, the central challenge lies not merely in identifying taxa that differ between groups but in determining whether a compact and interpretable microbial signature can distinguish NEC across cohorts with different clinical backgrounds. Conventional diversity metrics and ordination-based analyses may reveal group-level separation, but their performance is often limited in small or heterogeneous cohorts, particularly when inter-individual variation dominates the microbial landscape [4,10]. By contrast, differential abundance analyses frequently generate extensive lists of candidate genera, many of which are cohort-specific and difficult to prioritize for downstream validation. Reviews of NEC biomarkers in the machine learning era have noted that many proposed models perform well in internal validation but fail to generalize, largely because of confounding, feature instability, and overly optimistic evaluation strategies [14]. Explainable modeling frameworks may help bridge the gap between association and application by identifying a small set of taxa that captures the main predictive signal and by clarifying the direction of their risk contributions [8,9,14,15,16,17]. Yet in NEC microbiome research, it remains uncertain whether discrimination is driven primarily by global community restructuring or by a limited number of key taxa and how antibiotic exposure modifies this relationship. It also remains unclear whether antibiotic-associated perturbation weakens, mimics, or merely coexists with the NEC-associated microbial state. Resolving this issue requires analyses across multiple cohorts with contrasting group structures, including settings in which the effects of antibiotics and postnatal age can be separated from disease status.

This study aimed to determine whether a reproducible genus-level NEC signal could be identified across heterogeneous cohorts. We also examined whether this signal was related to antibiotic exposure and early development and retained in an independent real-world cohort. We therefore used a three-stage framework comprising discovery, contextual analysis, and external validation. By analyzing cohorts with different grouping structures separately and integrating them at the level of recurrent directional patterns, we sought to distinguish cross-cohort microbial features associated with NEC from cohort-specific variation. We further used machine learning-based feature prioritization to derive a compact and interpretable genus-level signature. This design allowed us to assess both the reproducibility and the practical limits of a reduced NEC-associated microbial panel.

## 2. Materials and Methods

### 2.1. Data Sources and Study Design

This study used a three-stage framework comprising discovery, contextual analysis, and external validation. Public datasets were analyzed separately rather than merged into a single pooled abundance matrix because sample composition, sequencing protocols, and available clinical annotations differed between cohorts. Cross-cohort integration was therefore performed at the level of recurrent genus-level directionality and signature consistency, not through direct batch-corrected pooling. The discovery cohort (NCBI Sequence Read Archive BioProject PRJNA998599; 36 NEC fecal samples, 67 non-NEC fecal samples) was used to identify NEC-associated genera and derive a reduced microbial signature. The contextual cohort (NCBI Sequence Read Archive BioProject PRJNA1238458; NEC *n* = 32 fecal samples, NEC_ABT *n* = 11 fecal samples, Control_P5 *n* = 15 fecal samples collected at postnatal day 5, and Control_P8 *n* = 15 fecal samples collected at postnatal day 8) was used to examine disease-, antibiotic-, and development-related microbial patterns. The independent real-world cohort was used only for external evaluation of the prespecified reduced signature. Because detailed patient-level covariates were not uniformly available across public datasets, cross-cohort comparisons were interpreted primarily at the level of conserved microbial patterns rather than fully adjusted pooled effect estimates (Supplementary Table S1).

### 2.2. Independent Real-World Cohort and Sample Collection

The validation cohort comprised preterm infants admitted to a single neonatal intensive care unit. Infants with a gestational age of less than 33 weeks and a birth weight greater than 950 g were eligible for inclusion. NEC cases were defined according to Bell stage II or III criteria, and infants without infectious complications were included as controls. In this validation setting, several major microbiome-related clinical exposures were relatively uniform, including delivery in the same center, cesarean delivery, formula feeding, routine prophylactic antibiotic exposure after NICU admission, and no probiotic administration during the study period. This reduced variation in several key confounders within the independent validation cohort. Written informed consent was obtained from the parents before enrollment, and the study protocol was approved by the institutional ethics committee. Only NEC and non-NEC control samples were included in the present validation analysis (4 NEC, 17 non-NEC).

Fecal sampling started from neonatal meconium and continued until discharge or death, whichever occurred first. Although daily collection was intended, the interval between 2 collections from the same infant did not exceed 7 days. Each stool sample was collected from the diaper with a sterile spatula within 30 min of defecation, transferred into cryogenic vials, placed immediately on dry ice, and stored at −80 °C within 30 min without additives. All samples were collected and stored before the final diagnosis of the respective infants was known.

### 2.3. Sample Processing and Sequencing

Following sample collection, fecal specimens were processed for microbial DNA extraction and genomic sequencing. Fecal samples were thawed on ice, and total microbial DNA was extracted using the QIAamp PowerFecal Pro DNA Kit (QIAGEN, Hilden, Germany) according to the manufacturer’s instructions. DNA concentration and purity were evaluated using the Qubit dsDNA HS Assay Kit (Thermo Fisher Scientific, Waltham, MA, USA) and NanoDrop One Spectrophotometer (Thermo Fisher Scientific, Waltham, MA, USA), respectively. DNA integrity was assessed before library preparation. Qualified DNA samples were used for library construction using the NEBNext Ultra II DNA Library Prep Kit (New England Biolabs, Ipswich, MA, USA), followed by paired-end sequencing on an Illumina NovaSeq 6000 sequencing platform according to standard procedures. Raw sequencing data were generated in FASTQ format and subsequently processed through quality control, host-derived read removal, and microbial taxonomic profiling workflows.

### 2.4. Raw Data Preprocessing

Raw paired-end sequencing reads were processed using fastp (v0.23.4) for quality control and adapter trimming. Reads with a mean Phred quality score < 20, reads containing > 5 ambiguous bases, and reads shorter than 50 bp after trimming were removed. Automatic adapter detection was applied to paired-end reads, and identical preprocessing settings were used for all samples in a multi-threaded workflow. Host-derived contamination was then removed by aligning quality-filtered reads to the human reference genome (hg38) with Bowtie2 (v2.5.1; --very-sensitive). Only non-host reads were retained for downstream taxonomic annotation and abundance profiling.

### 2.5. Taxonomic Annotation and Abundance Matrix Construction

Taxonomic annotation was performed on host-depleted reads, and all downstream analyses were conducted at the genus level. For each sample, genus-level read counts were summarized into a count matrix, and relative abundance profiles were calculated by normalizing each genus-level read count to the total reads reported in the corresponding Kraken2 report. Zero counts were retained to preserve matrix integrity. To ensure consistent feature definitions across datasets, abundance matrices from different cohorts were harmonized using a unified genus reference. Genera not detected in a given sample or cohort were recorded as absent and assigned a value of zero.

### 2.6. Low-Information Feature Filtering

To reduce sparsity and noise from low-abundance taxa, genus-level features were filtered. Genera were retained only if they met both of the following criteria: presence in at least 10% of samples and a maximum relative abundance of at least 0.1% (0.001) in at least one sample. The numbers of genera before and after filtering were recorded for each cohort to document the effect of feature reduction. For machine learning analyses, low-information filtering was performed within the discovery cohort before model development, and the independent validation cohort was used only for evaluation of the prespecified reduced signature.

### 2.7. Diversity Analysis

Alpha diversity was evaluated using the Shannon index and observed genus richness, with group comparisons performed by the Wilcoxon rank-sum test. Beta diversity was assessed using Bray–Curtis dissimilarity and visualized by principal coordinates analysis (PCoA). Differences in community composition between groups were tested by permutational multivariate analysis of variance (PERMANOVA). To minimize spurious PERMANOVA findings attributable to unequal within-group dispersion, homogeneity of multivariate dispersion was examined using the betadisper test.

### 2.8. Differential Abundance Analysis

Differential abundance at the genus level was evaluated using two complementary methods. LEfSe was used to identify taxa showing consistent between-group differences under predefined significance thresholds and linear discriminant analysis (LDA) effect-size criteria. MaAsLin2 was applied in parallel to model the associations between genus-level relative abundance and group status, providing regression coefficients and the corresponding direction of effect.

### 2.9. Machine Learning and Model Interpretability

Machine learning analyses were conducted exclusively in the discovery cohort and were intended primarily for feature prioritization and construction of a reduced microbial signature. Genus-level relative abundance features retained after low-information filtering were used as candidate predictors, and the group outcome was encoded as non-NEC or NEC. For model training and feature-ranking analyses, the discovery cohort was randomly divided into training and testing subsets at a 70:30 ratio using stratified sampling, with a fixed random seed to preserve the group distribution. Feature values were centered and scaled using parameters estimated from the training subset, and the same transformation was applied to the testing subset.

Baseline logistic regression, random forest, and extreme gradient boosting (XGBoost) models were fitted using the genus-level features. The random forest model contained 500 trees, and feature importance was quantified using the mean decrease in Gini impurity. The XGBoost model used a binary logistic objective and AUC as the evaluation metric, with a maximum tree depth of 4, a learning rate of 0.1, subsample and column-sampling fractions of 0.8, and 200 boosting rounds. XGBoost feature importance was ranked according to gain. SHapley Additive exPlanations (SHAP) values were calculated from the fitted XGBoost model to assess the direction and relative contribution of individual genera to model output.

Taxa consistently prioritized across the random forest and XGBoost rankings, together with their biological direction in the NEC-associated abundance pattern, were retained as candidate signature genera. Their standardized relative abundances were entered into a multivariable logistic regression model to construct the reduced microbial signature, and the fitted coefficients were used to describe the direction of contribution of each genus. Internal model performance and stability were assessed by stratified 10-fold cross-validation within the discovery cohort, with AUC as the primary metric and accuracy, sensitivity, specificity, and F1 score calculated as secondary measures using a probability threshold of 0.5. The independent validation cohort was kept separate throughout model development and was not used for feature selection, preprocessing parameter estimation, coefficient fitting, hyperparameter selection, or model tuning.

### 2.10. Risk Index Construction and External Validation

A microbial risk index was developed from genus-level features and their corresponding logistic regression coefficients. Sample-level risk scores were calculated by weighting relative abundances with the model-derived coefficients. Genera with odds ratios greater than 1 were defined as risk-associated, whereas those with odds ratios less than 1 were defined as protective. Internal performance of the risk index was assessed by comparing receiver operating characteristic curves and cross-validated AUC values between the full model and the reduced model based on selected core genera. External validation was performed in the independent real-world cohort. Risk scores were then calculated directly, and discriminatory performance was evaluated by AUC.

### 2.11. Statistical Analysis

All statistical analyses were performed in R. Group comparisons for alpha diversity were conducted using nonparametric methods. For beta diversity, statistical significance was assessed with permutation-based testing. For differential abundance, significance was determined according to the prespecified criteria of each analytical method, with false discovery rate (FDR) correction applied. Effect estimates and their directions were reported together with the corresponding statistical measures. For machine learning analyses, model performance was evaluated primarily by AUC, and cross-validation was used to assess model stability and limit overfitting. All statistical tests were two-sided. Where applicable, exact *p*-values, effect estimates (e.g., log2 fold change, odds ratio, or PERMANOVA R^2^), and 95% confidence intervals are reported.

## 3. Results

### 3.1. Initial Microbiota Shifts in the NEC Discovery Cohort

**Figure 1:**
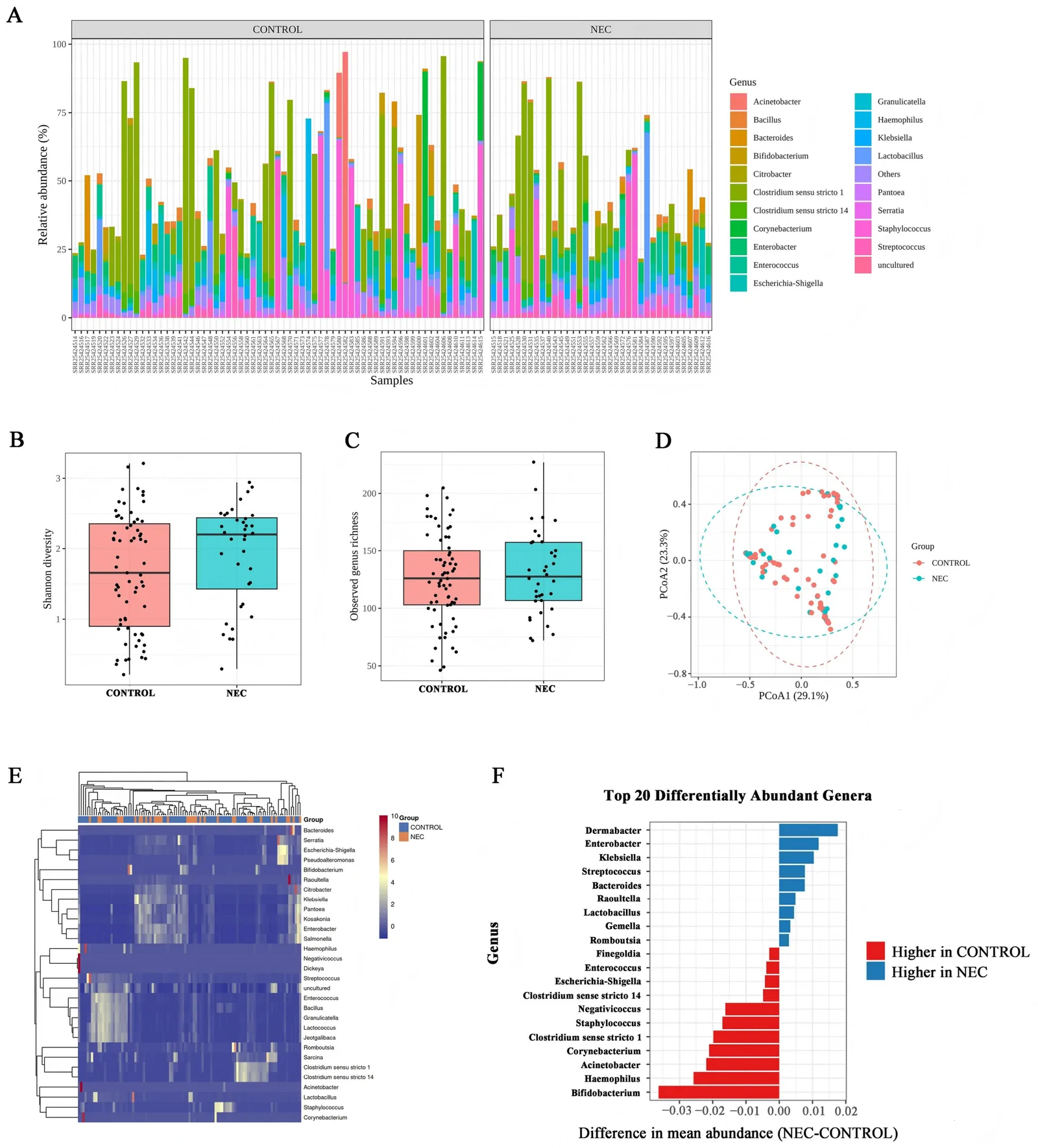
Initial microbiota alterations and genus-level compositional shifts in the NEC discovery cohort: (**A**) Relative abundance of major genera in control and NEC samples. (**B**) Shannon diversity. (**C**) Observed genus richness. (**D**) Bray–Curtis PCoA of control and NEC samples. (**E**) Heatmap of genus-level abundance profiles. (**F**) Top 20 differentially abundant genera between the two groups.

In the discovery cohort, genus-level profiling showed an altered microbial composition in NEC samples relative to controls, although overall community structure remained heterogeneous across individuals (Figure 1A). Alpha-diversity analysis indicated that Shannon diversity and observed genus richness were both slightly higher in the NEC group, indicating higher within-sample diversity in this cohort, although the magnitude of the difference was limited (Figure 1B,C). Bray–Curtis PCoA demonstrated only partial separation between NEC and control samples, with substantial overlap between groups, indicating that microbial differences in this cohort were present but not fully resolved by global diversity metrics alone (Figure 1D).

At the taxonomic level, NEC samples nonetheless showed a clear directional redistribution of key genera. NEC samples tended to cluster around a distinct abundance pattern involving several dominant taxa, supporting genus-level compositional remodeling (Figure 1E). Differential abundance ranking showed relative enrichment of *Enterobacter*, *Klebsiella*, *Streptococcus*, and *Raoultella* in NEC, whereas controls showed higher relative abundance of *Bifidobacterium*, *Haemophilus*, *Acinetobacter*, *Corynebacterium*, and *Clostridium sensu stricto 1* (Figure 1F). The early microbial signal in the discovery cohort was defined less by a coherent global shift than by selective genus-level reorganization, characterized by expansion of potential opportunistic taxa and reduction in taxa associated with microbial homeostasis.

### 3.2. Identification of a Compact NEC Microbial Signature

**Figure 2:**
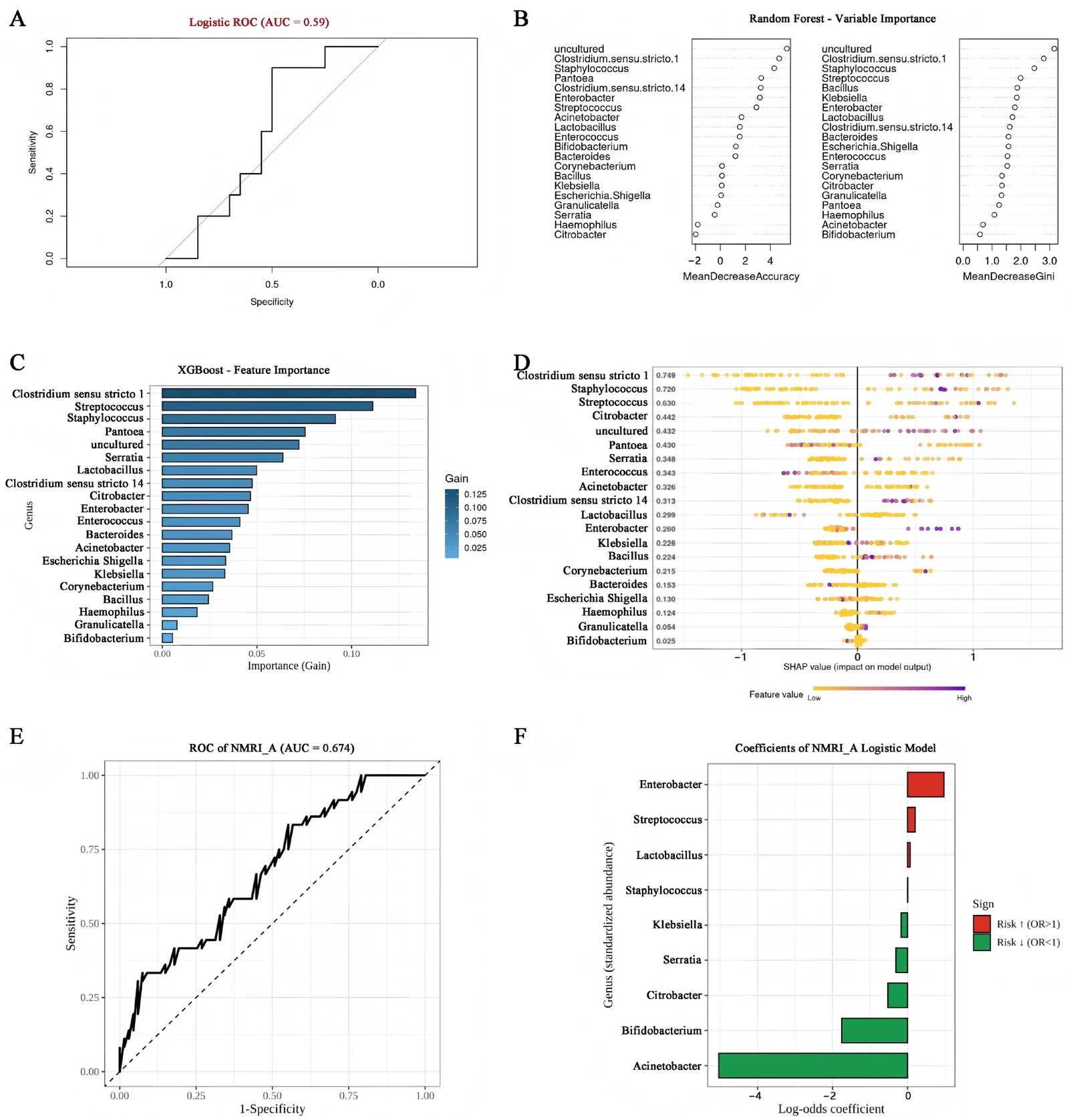
Machine learning identification of a compact NEC-associated microbial signature: (**A**) ROC curve of the baseline logistic model using the initial genus set. (**B**) Random forest variable importance. (**C**) XGBoost feature importance. (**D**) SHAP summary plot of key genera. (**E**) ROC curve of the reduced logistic signature model. (**F**) Coefficients of genera included in the final compact model. ↑: Higher odds of NMRI_A, ↓: Lower odds of NMRI_A.

Because the discovery cohort showed clearer genus-level redistribution than whole-community separation, we next used machine learning methods to prioritize a smaller set of informative genera. Using the complete genus set as input, the baseline logistic model showed modest discriminatory ability, with an AUC of 0.59, indicating limited classification performance when the unfiltered microbial profile was used directly (Figure 2A). To refine these features, we compared taxon rankings across multiple machine learning methods. Random forest identified a group of highly contributing genera, including *uncultured taxa*, *Clostridium sensu stricto 1*, *Staphylococcus*, *Streptococcus*, and *Bacillus*, while also retaining established NEC-associated genera such as *Enterobacter*, *Klebsiella*, and *Citrobacter* among the more relevant variables (Figure 2B). XGBoost prioritized a partially overlapping but biologically coherent set of taxa, with *Clostridium sensu stricto 1*, *Streptococcus*, *Staphylococcus*, *Pantoea*, *Serratia*, *Lactobacillus*, *Citrobacter*, and *Enterobacter* ranking among the leading contributors (Figure 2C). SHAP analysis showed that these features contributed consistently to model output and indicated that the NEC-associated signal arose from the combined effect of several key genera rather than from a single dominant taxon (Figure 2D).

On the basis of taxa that recurred across models, we constructed a reduced logistic signature. This compact model improved discriminatory performance to an AUC of 0.674, supporting the value of feature prioritization over direct use of the full microbial profile (Figure 2E). In the final model, *Enterobacter* and *Streptococcus* had positive coefficients, whereas Acinetobacter and Bifidobacterium contributed in the opposite direction, with additional genera providing smaller complementary effects (Figure 2F). These findings indicate that feature prioritization can derive a smaller NEC-associated genus panel from a heterogeneous feature space.

### 3.3. Microbiota Divergence Across Disease, Antibiotics, and Early Development

**Figure 3:**
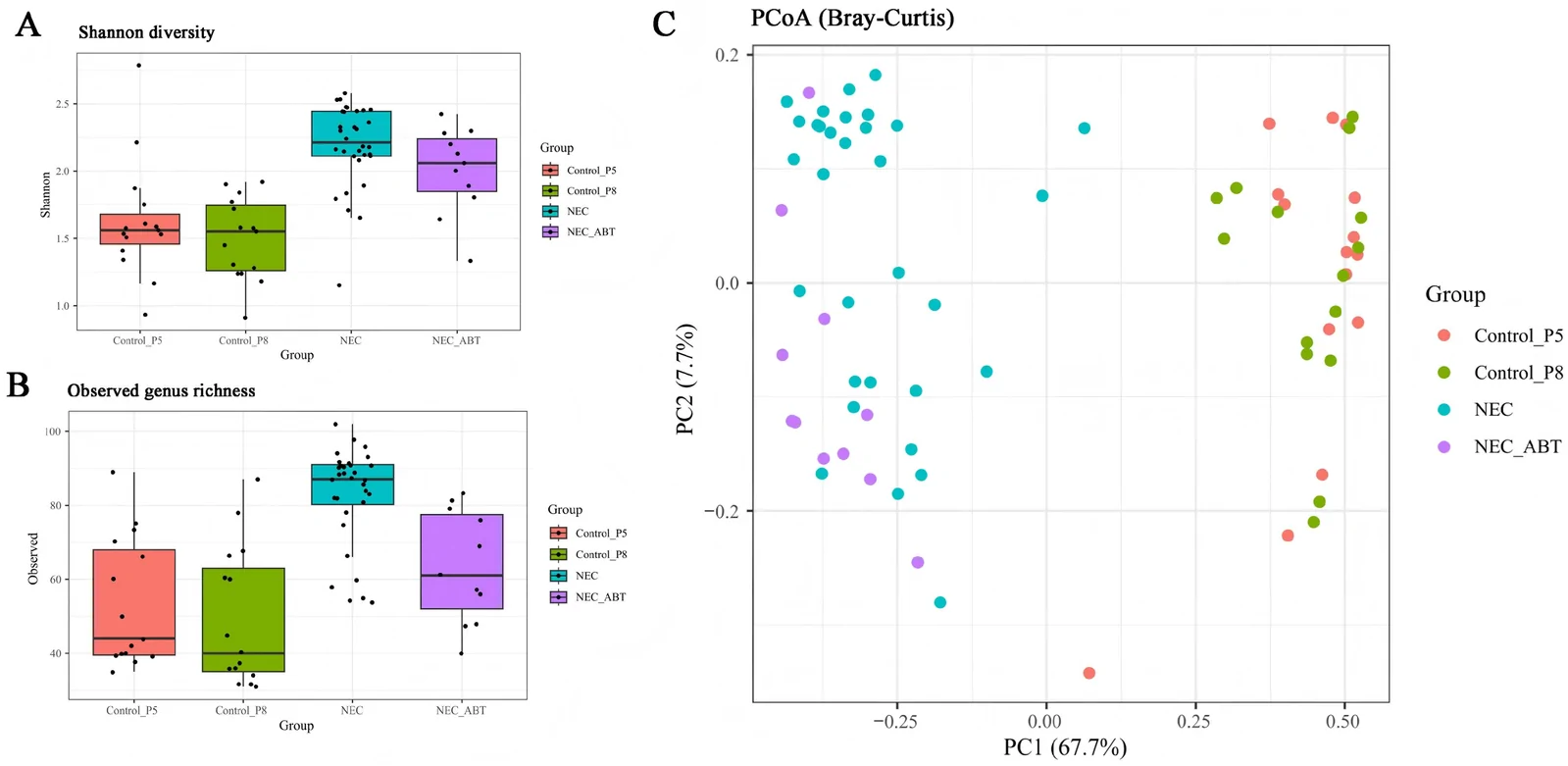
Microbiota divergence across disease status, antibiotic exposure, and early developmental stage in the four-group cohort: (**A**) Shannon diversity across Control_P5, Control_P8, NEC, and NEC_ABT groups. (**B**) Observed genus richness across the four groups. (**C**) Bray–Curtis PCoA showing community-level separation among control, NEC, and antibiotic-exposed NEC samples.

To further define the microbial context of NEC-associated dysbiosis, we analyzed an independent four-group cohort comprising Control_P5, Control_P8, NEC, and NEC_ABT samples. At the alpha-diversity level, Shannon diversity and observed genus richness showed a graded distribution across groups. The NEC group had the highest diversity and richness, whereas both control groups remained at lower levels. The NEC_ABT group showed intermediate values between untreated NEC and controls, consistent with partial modification of the NEC-associated microbiota in the setting of antibiotic exposure but not restoration to the control state (Figure 3A,B). Group differences were more clearly resolved by beta-diversity analysis. Bray–Curtis PCoA showed evident separation between NEC-related samples and controls along the primary coordinate, whereas Control_P5 and Control_P8 clustered close to one another, indicating relative continuity during early healthy development (Figure 3C). NEC samples formed a distinct cluster apart from both control groups, while NEC_ABT samples remained closer to NEC than to either control cluster, consistent with persistent disease-related community restructuring despite antibiotic exposure. The four-group cohort showed microbial divergence between NEC-related samples and healthy controls, while NEC_ABT samples remained distinct from both control groups.

### 3.4. NEC-Associated Genus Redistribution Favors Opportunistic Taxa

**Figure 4:**
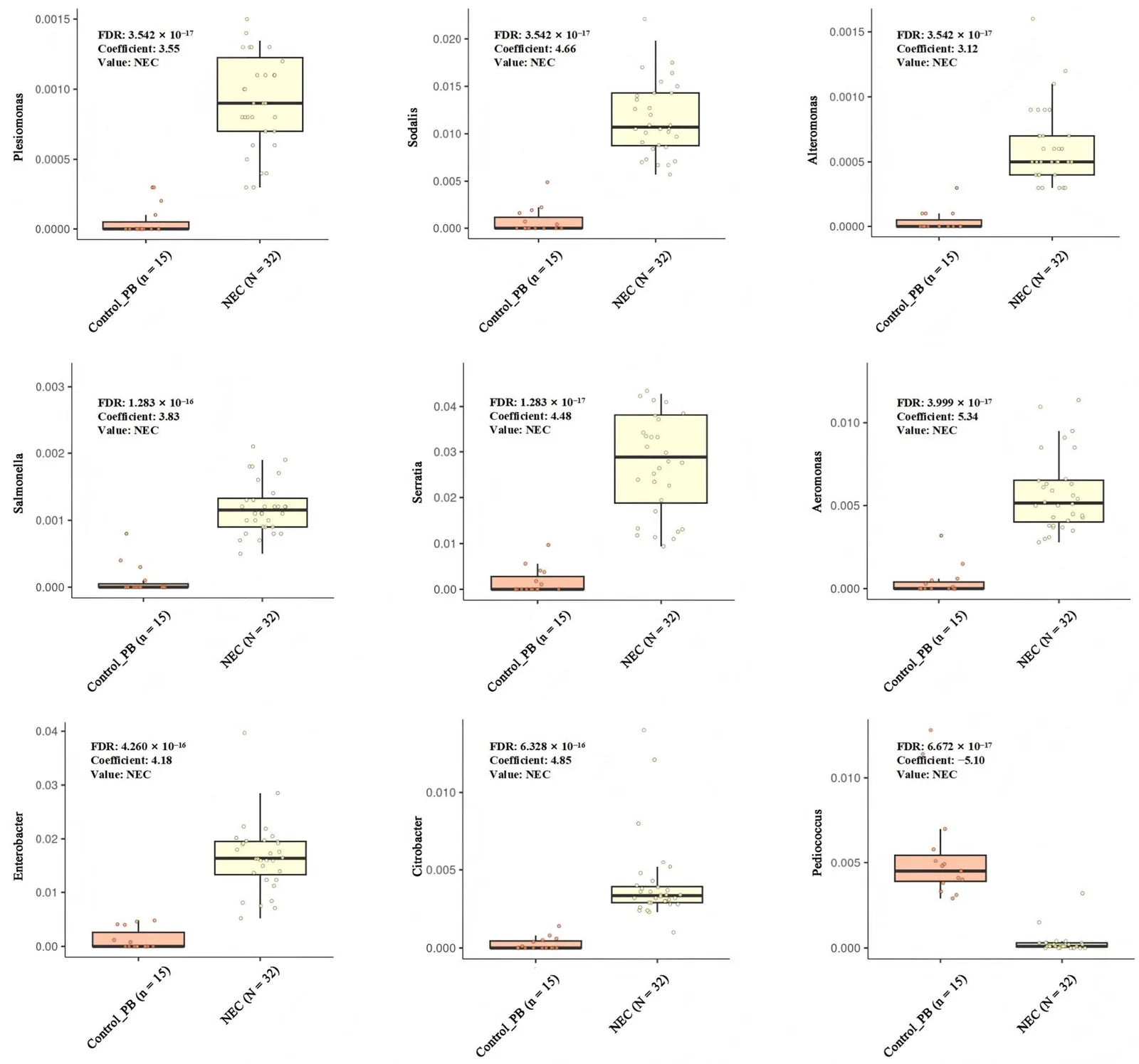
NEC-associated genus redistribution in the four-group cohort.

To define the taxonomic basis of the community shift observed in the four-group cohort, we then examined genus-level differences between NEC and the age-matched healthy group. This comparison showed that the NEC microbiota was characterized by directional redistribution of multiple taxa rather than fluctuation of a single dominant genus. Several genera with opportunistic or potentially pathogenic features were markedly enriched in NEC, including *Plesiomonas*, *Sodalis*, *Alteromonas*, *Salmonella*, *Serratia*, *Aeromonas*, *Enterobacter*, and *Citrobacter*, all of which showed clear increases in relative abundance compared with controls (Figure 4). The concordant increase across these genera indicates that NEC was associated with broad taxonomic reorganization toward a pathogen-enriched configuration. The taxa associated with a more stable microbial background were reduced in NEC. *Pediococcus* exhibited a marked decrease relative to controls, in contrast to the coordinated expansion of the opportunistic taxa described above. The NEC-associated microbial pattern in this cohort involved enrichment of several dysbiosis-related genera and depletion of taxa associated with microbial homeostasis, beyond the differences observed in diversity metrics alone.

### 3.5. Antibiotic Exposure Reshapes but Does Not Normalize the NEC Microbiota

**Figure 5:**
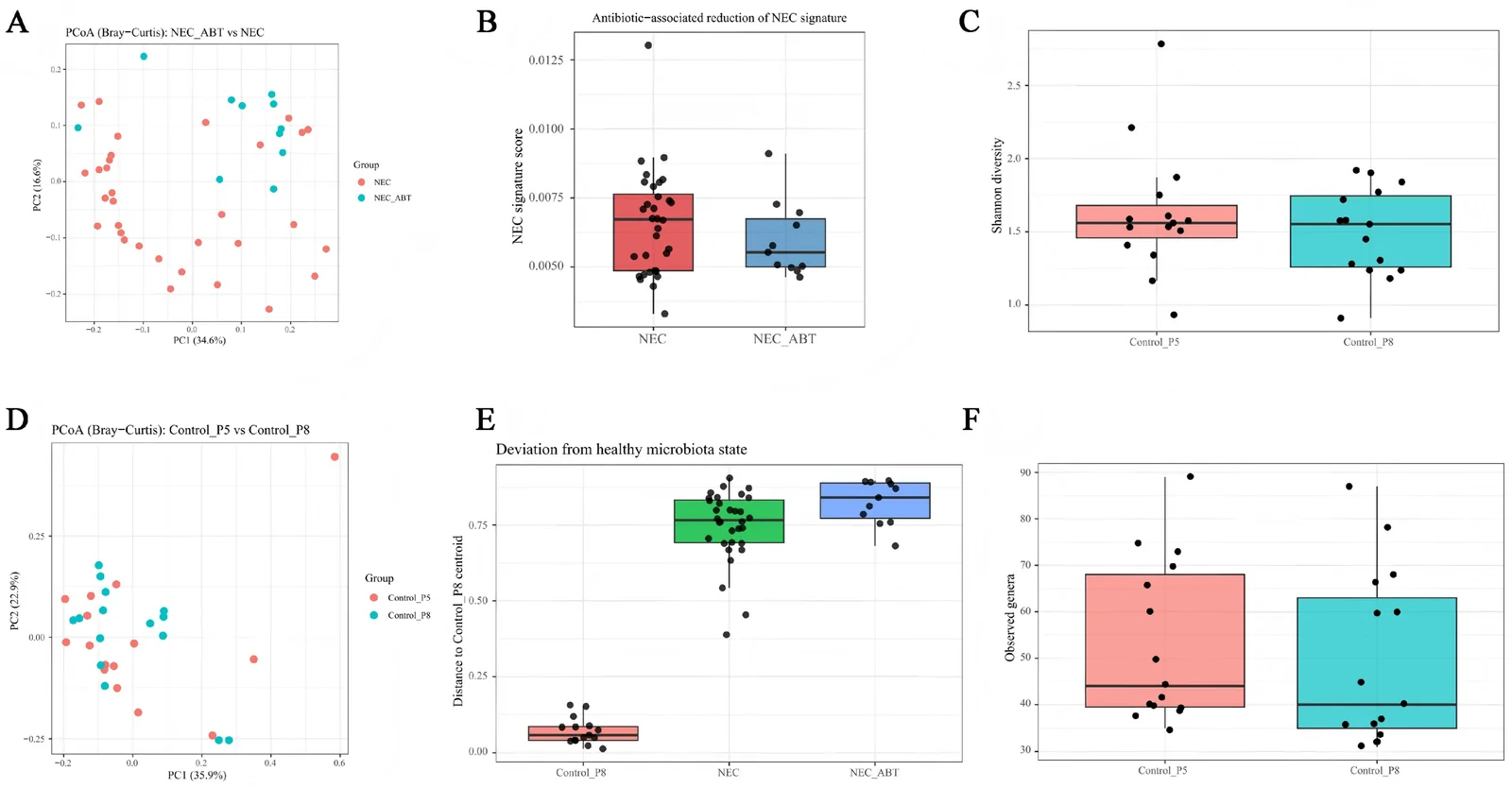
Antibiotic exposure reshapes the NEC microbiota but does not restore the healthy developmental state: (**A**) Bray–Curtis PCoA comparing NEC and NEC_ABT samples. (**B**) NEC dysbiosis signature score in untreated and antibiotic-exposed NEC samples. (**C**) Shannon diversity between Control_P5 and Control_P8. (**D**) Bray–Curtis PCoA of Control_P5 and Control_P8 samples. (**E**) Distance from the healthy microbiota state defined by the Control_P8 centroid. (**F**) Observed genus richness between Control_P5 and Control_P8.

We compared untreated NEC samples with antibiotic-exposed NEC samples in the four-group cohort. Bray–Curtis PCoA showed partial redistribution of NEC_ABT samples relative to untreated NEC, indicating that the microbiota differed between the two groups (Figure 5A). The NEC dysbiosis signature score was lower in NEC_ABT than in untreated NEC (Figure 5B). NEC_ABT samples, nevertheless, remained closer to the NEC state than to the healthy control state (Figure 5A,B). These findings indicate partial attenuation of the NEC-associated signature in the setting of antibiotic exposure but do not establish a causal normalizing or protective effect of antibiotics.

We then examined whether NEC-associated dysbiosis could be interpreted as an extension of normal early microbial development. In contrast to the marked disease-related divergence described above, Shannon diversity and observed genus richness were broadly similar between Control_P5 and Control_P8, indicating relative stability of alpha-diversity metrics across this early, healthy developmental window (Figure 5C,F). Beta-diversity analysis likewise showed substantial overlap between the two control groups, with only limited separation on Bray–Curtis PCoA, supporting continuity of the normal developmental trajectory from P5 to P8 rather than a major ecological transition (Figure 5D). Both NEC and NEC_ABT samples showed clear displacement from the healthy microbiota state when distance to the Control_P8 centroid was quantified, and neither group approached the low-distance pattern observed in healthy controls (Figure 5E). Antibiotic exposure may dampen the NEC-associated microbial signature but does not restore the microbiota to the range observed during normal early development. Within the limits of this cohort, the NEC microbial configuration appeared more consistent with a disease-related deviation from normal early development.

### 3.6. In-House Evaluation of the NEC-Associated Dysbiosis Pattern

**Figure 6:**
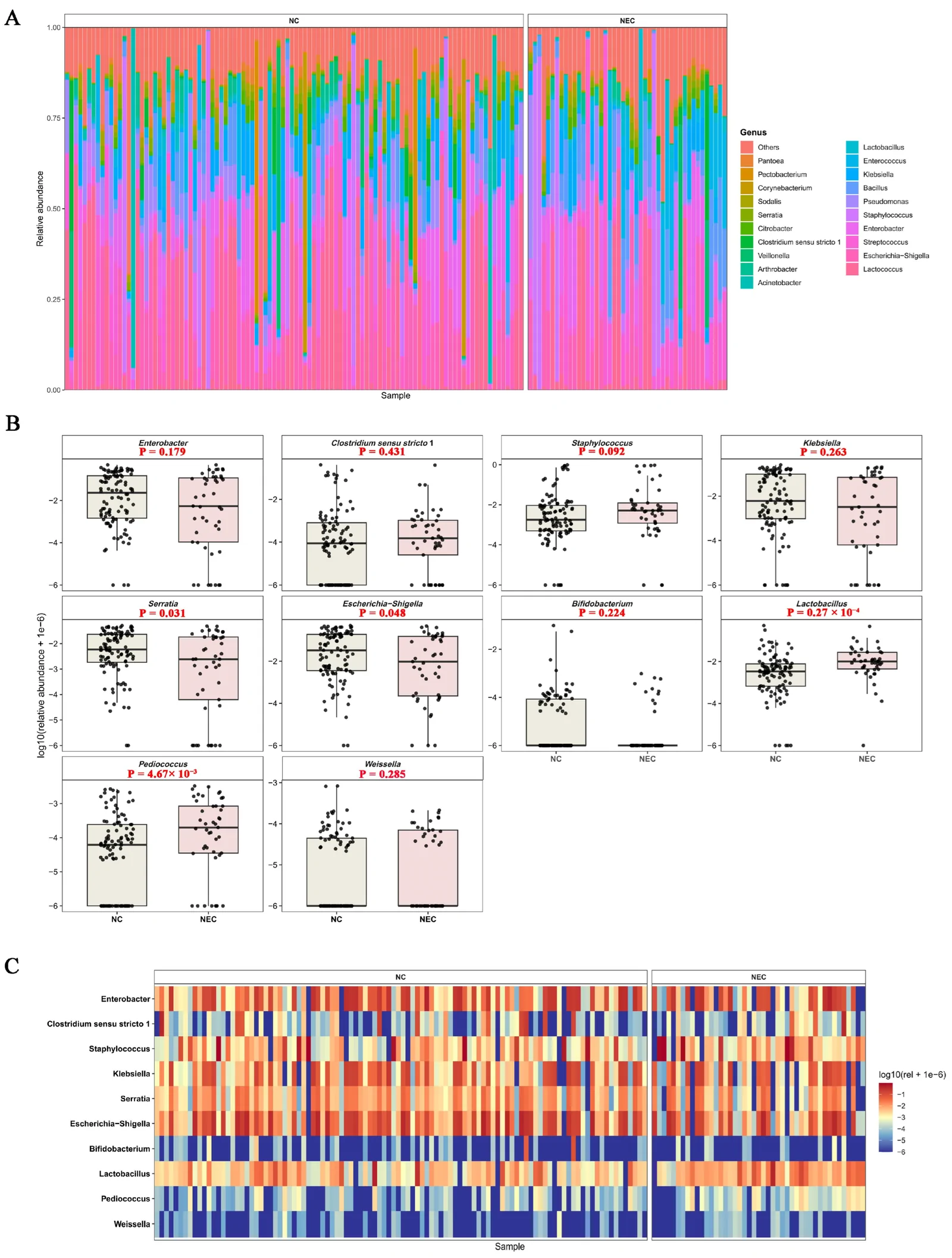
Validation of the core NEC-associated genera in collected samples: (**A**) Relative abundance of major genera in NC and NEC samples. (**B**) Groupwise comparison of prespecified core genera between NC and NEC. (**C**) Heatmap showing the abundance pattern of the core NEC-associated genus panel across samples.

To further assess the robustness of the NEC-associated microbial pattern identified in the public cohorts, we analyzed our collected samples comprising NC and NEC groups. Stacked bar plots showed substantial inter-individual heterogeneity in both groups (Figure 6A). Several dominant genera, nevertheless, differed between NC and NEC, indicating that the NEC-associated signal remained detectable at the group level. Evaluation of the prespecified core genera showed that our collected samples retained the same direction for several taxa identified in the discovery analysis. In particular, Serratia and Escherichia–Shigella were more abundant in NEC, whereas Lactobacillus and Pediococcus were relatively more abundant in NC, showing partial agreement with the directional pattern observed in the discovery analysis (Figure 6B). Although the magnitude of difference varied among genera, the core panel showed a group-related abundance pattern that was more apparent at the combined-panel level than for any single taxon (Figure 6C).

**Figure 7:**
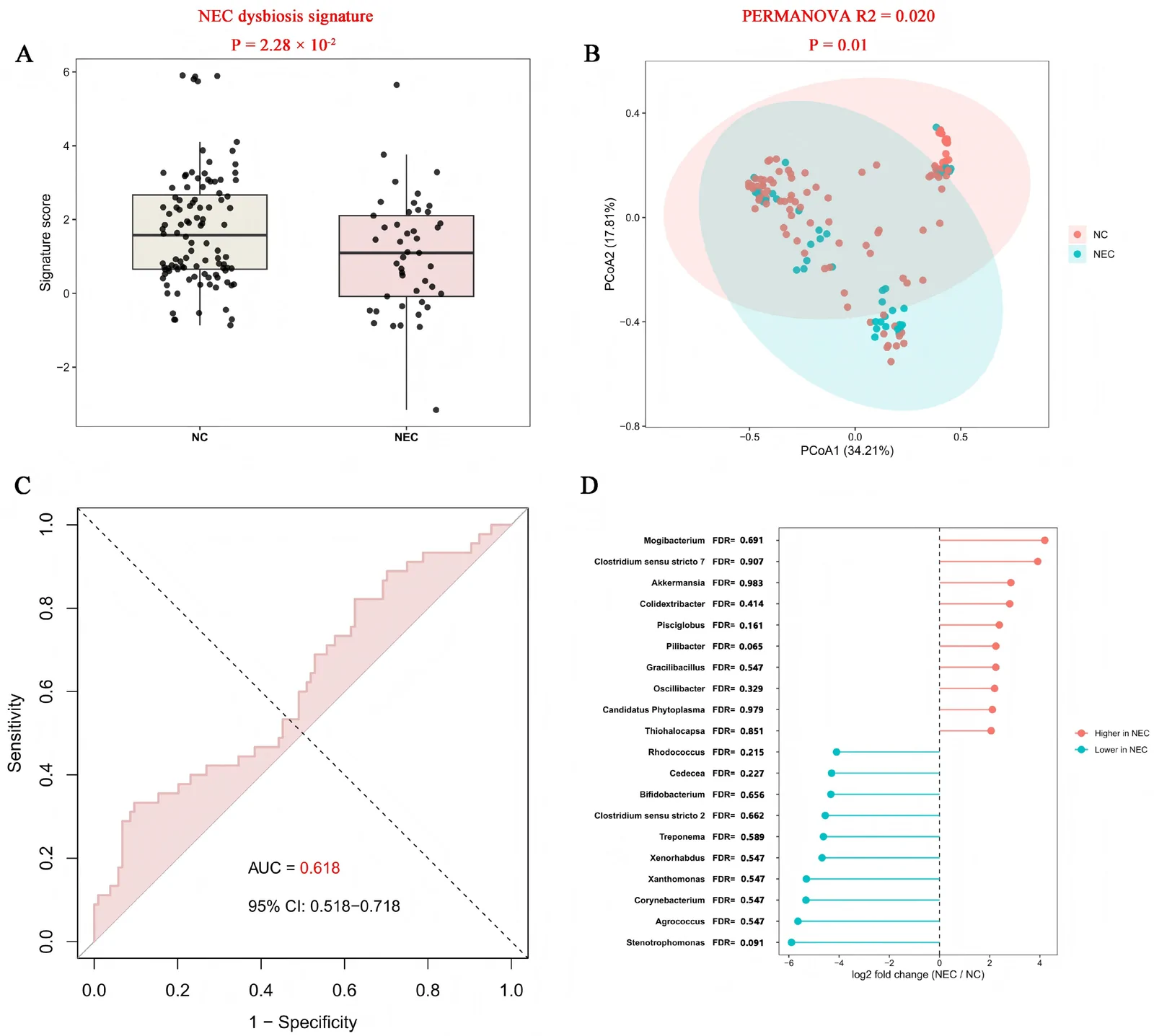
Composite signature and community-level validation in collected samples: (**A**) NEC dysbiosis signature score in NC and NEC samples. (**B**) Bray–Curtis PCoA comparing NC and NEC. (**C**) ROC curve of the composite signature in our collected samples. (**D**) Differentially abundant genera identified between NC and NEC.

We next examined whether this coordinated genus-level pattern could be translated into a composite microbial signal in our collected samples. The NEC dysbiosis signature score differed significantly between NC and NEC, indicating that the reduced signature retained limited discriminatory information in this sample set (Figure 7A). Beta-diversity analysis likewise showed significant community-level separation between the two groups (PERMANOVA R^2^ = 0.020, *p* = 0.01; Figure 7B). ROC analysis yielded an AUC of 0.618, indicating modest discriminatory performance of the microbial signature in the independent sample set (Figure 7C). Differential abundance analysis further identified additional NEC-associated and NC-associated genera, extending the observed dysbiosis pattern beyond the predefined core panel while remaining broadly consistent with the ecological imbalance between opportunistic and commensal taxa (Figure 7D).

## 4. Discussion

NEC remains one of the most severe gastrointestinal complications of prematurity. Its pathogenesis is now understood as the result of converging factors, including intestinal immaturity, exaggerated innate immune activation, enteral exposure, and abnormal microbial colonization, rather than the effect of a single causative pathogen [18,19,20]. Across heterogeneous datasets, recurrent genus-level directional changes were more consistent than shifts in global diversity metrics. This observation showed partial cross-cohort agreement between the public datasets and our collected samples and agrees with prior studies showing that microbiota alterations may precede overt NEC [21,22,23]. It is also consistent with the meta-analysis by Pammi, in which preterm infants who later developed NEC showed enrichment of Proteobacteria, whereas alpha- and beta-diversity findings were not stable across studies [10]. The heterogeneity observed in the discovery analyses may reflect biological and clinical variability in NEC while allowing recurrent genus-level associations to remain detectable.

The slightly higher Shannon diversity and observed genus richness in the NEC group differed from studies reporting reduced alpha diversity in infants with NEC. This discrepancy may reflect differences in sampling time relative to disease onset, postnatal age, gestational maturity, feeding practice, antibiotic exposure, and clinical management across cohorts. Methodological variation, including sequencing strategy, taxonomic resolution, preprocessing procedures, feature filtering, and the diversity indices used, may also contribute to differences between studies. In our cohort, the higher alpha-diversity values occurred together with enrichment of opportunistic genera and depletion of commensal or probiotic-associated taxa, indicating that within-sample diversity alone did not capture the direction or clinical relevance of microbial restructuring.

A recurrent finding in our study was enrichment of opportunistic or environment-associated genera [24,25], including *Enterobacter*, *Klebsiella*, *Serratia*, and *Escherichia–Shigella*, together with depletion of commensal or probiotic-associated taxa such as *Bifidobacterium* and *Lactobacillus*. NEC is often preceded by overrepresentation of Enterobacteriaceae and other Gram-negative organisms with inflammatory potential. Ward et al. showed at strain resolution that colonization by uropathogenic *Escherichia coli* was associated with both NEC and mortality in extremely preterm infants [26]. Olm et al. further reported increased bacterial replication, Klebsiella expansion, and enrichment of fimbriae-encoding organisms before NEC onset [16]. Sim et al. described both Clostridium-associated and Klebsiella-associated prediagnostic patterns, suggesting that NEC may arise through more than one microbial route while still converging on a shared dysbiotic state. These findings suggest that NEC may be associated with recurrent directional changes in microbial composition rather than a single fixed taxonomic profile.

Our analyses also place this dysbiosis pattern within the framework of early microbial development [27,28,29,30,31]. La Rosa et al. demonstrated that the preterm gut follows an ordered succession from Bacilli to Gammaproteobacteria to Clostridia [32], and Shen et al. showed that postnatal age is more strongly associated with early microbiome dynamics than gestational age [33]. In our four-group cohort, the limited separation between Control_P5 and Control_P8 contrasted with the displacement of NEC and NEC_ABT samples, suggesting that NEC-associated microbial alterations may differ from the changes observed during this early developmental interval. The NEC_ABT group showed attenuation of the NEC-associated signature but did not return toward the control configuration. This pattern is compatible with microbiota reshaping in the setting of antibiotic exposure [34,35,36,37,38]. Although the association between early antibiotic exposure and NEC is not fully consistent across studies, prolonged or unnecessary empirical antibiotic use has repeatedly been linked to adverse outcomes and remains a plausible contributor to dysbiosis in preterm infants [7,37,38,39,40,41].

Another point of this study is that machine learning-based feature prioritization was used to derive a reduced and interpretable NEC-associated microbial panel from a heterogeneous feature space [42,43,44,45]. Traditional diversity indices and unfiltered abundance matrices often have limited sensitivity in NEC research, particularly when sample size is modest and microbial configurations vary across cohorts [46,47,48]. Earlier studies showed that microbial features preceding NEC may carry predictive information, and more recent work has applied machine learning frameworks to microbiome data [15,49]. Our findings extend this line of work by showing that feature prioritization can derive a smaller panel characterized by enrichment of opportunistic taxa and depletion of commensal or probiotic-associated genera.

This study also has several limitations. First, detailed clinical covariates were not uniformly available across the public datasets, and residual confounding by gestational maturity, feeding practice, delivery mode, probiotic exposure, and treatment-related factors cannot be fully excluded. The detailed antibiotic exposure information, including antibiotic classes and treatment duration in the NEC-ATB group, was not available from the public dataset, limiting further evaluation of the specific effects of different antibiotic regimens on gut microbiota alterations. This concern was partly mitigated in the independent validation cohort because samples were collected in a single NICU under a relatively standardized early-care protocol, but it was not eliminated in the cross-cohort analyses. Second, the independent validation cohort was relatively small (4 NEC and 17 non-NEC fecal samples), which limited the statistical robustness and generalizability of the external validation results. Although the reduced microbial signature showed consistent directional changes across cohorts, its discriminatory performance remained moderate in our independent cohort, indicating that its current role is more supportive than diagnostic and therefore not suitable for clinical implementation. Validation in larger independent neonatal cohorts will be necessary before potential clinical application can be considered. Third, this study was based on cross-cohort comparative analysis and lacked longitudinal multi-omics data or mechanistic validation. The biological roles of the identified taxa in NEC progression therefore require further confirmation.

Despite these limitations, this study identifies several genus-level microbial alterations that recurred across independent NEC cohorts. The conserved microbial features identified in this study may provide candidate targets for future NEC risk assessment when integrated with clinical information and longitudinal monitoring. The current microbial signature requires further validation before clinical application, particularly in larger prospective neonatal cohorts. Future studies should incorporate detailed clinical variables, including antibiotic exposure and nutritional factors, together with longitudinal multi-omics analyses and experimental validation to determine the temporal relationship between microbial changes and NEC development.

## 5. Conclusions

This study found that NEC-associated gut microbiota showed greater cross-cohort agreement at the level of selected genus-level directional changes than in global community metrics. By integrating public cohorts with our collected samples, we derived a compact NEC-associated microbial signature that showed partial directional agreement across datasets and modest discriminatory performance. In the contextual cohort, antibiotic-exposed NEC samples showed a lower dysbiosis score than untreated NEC samples but remained distinct from healthy controls, while NEC-related samples also differed from the early control groups. The identified signature should therefore be considered an exploratory microbial biomarker and a candidate feature set for validation in larger longitudinal cohorts and mechanistic studies.

## Data Availability

The datasets used and analyzed during the current study are available from the corresponding author upon reasonable request.

## Supplementary Materials

The following supporting information can be downloaded at: https://www.mdpi.com/article/10.3390/pathogens15080785/s1, Table S1: Characteristics of the publicly available neonatal gut microbiome datasets included in the study.
