# Supplementary material for: Compact Gut Microbial Dysbiosis Signature Associated with Necrotizing Enterocolitis and Altered Early Microbiota Development
Source: Pathogens. 2026 Jul 24;15(8):785. doi: 10.3390/pathogens15080785 (PMC13516161; doi:10.3390/pathogens15080785)
Supplement: Supplementary file 1 [file pathogens-15-00785-s001.zip › pathogens-4397419-supplementary.pdf]

Supplementary Table S1. Characteristics of the publicly available neonatal gut microbiome datasets included in the study

**Characteristic**	**PRJNA998599**	**PRJNA1238458**
Role in analysis	Discovery cohort	Contextual animal cohort
Study population/host	Preterm infants (*Homo sapiens*)	Preterm infants (*Homo sapiens*)
Country/setting	France; prospective multicenter case-control study conducted in 20 NICUs	China; experimental dataset submitted by Children's Hospital of Fudan University
Samples analyzed	103 samples: 36 NEC and 67 controls	73 samples: 32 NEC, 11 NEC_ABT, 15 Control_P5, and 15 Control_P8
Sample type	fecal samples	fecal samples
Sequencing	16S rRNA V3-V4 amplicon sequencing; Illumina MiSeq; paired-end	16S rRNA amplicon sequencing; Illumina MiSeq; paired-end
Gestational/postnatal age	All enrolled infants were < 32 gestational weeks; median gestational age was 29 weeks (IQR 26-30) in both source-study groups*	Gestational age NA
Feeding practice	Human milk feeding was reported in 73% of NEC cases and 87% of controls*	NR
Available clinical/experimental variables	Public SRA metadata: NEC/control label and technical sequencing fields only	Experimental group, collection date, host species, sample origin, and sequencing fields; no individual-level clinical variables were reported
